# Small-Sided Games versus Continuous Endurance Training in Female Handball Players

**DOI:** 10.5114/jhk/163070

**Published:** 2023-07-15

**Authors:** Jan Bělka, Karel Hůlka, Michal Šafář

**Affiliations:** 1 Department of Sport, Faculty of Physical Culture, Palacky University Olomouc, Olomouc, Czech Republic.; 2 Department of Social Sciences in Kinanthropology, Faculty of Physical Culture, Palacky University Olomouc, Olomouc, Czech Republic.

**Keywords:** team handball, performance, specific fitness, testing

## Abstract

The main aim of the study was to compare the training methods of continuous endurance training versus handball small-sided games (SSGs) in female handball players during the preseason. Eighteen female handball players from a first league in the Czech Republic voluntarily participated in the study. They were divided into two groups (the SSG group [n = 9; age: 21.22 ± 3.03 years] and a running group (RG) [n = 9; age: 23.78 ± 3.77 years]). Both groups continued regular preseason training for six weeks. The SSG group played two small-sided games per week (a total of 12 games during the monitoring period) in their training sessions during the monitored six-week period. The RG ran twice per week, participating in continuous endurance training in the 12 training sessions during the monitored six-week period. Fitness tests (pretest/posttest) were performed prior to the six weeks of intervention and one week afterwards. The order of the fitness tests in sequence was as follows: 20-m and 30-m sprints, a Modified Agility T test (MAT), a Standing Long-Jump Test, ball-throwing velocity, and a Yo-Yo Intermittent Test level 1. In the SSG group, there was a significant improvement in the T test (p = 0.034), ball-throwing velocity (p = 0.019), and the Yo-Yo IR1 (p = 0.003) performance. The RG showed a significant improvement in the Standing Long-Jump Test (p = 0.049), ball-throwing velocity (p = 0.003), and the Yo-Yo IR1 (p = 0.003) performance. In conclusion, the SSGT method had a positive effect on female players’ MAT, ball-throwing velocity and YoYo IR1 results, and the CERT method had a significant effect only on the YoYo IR1 results. Handball SSGs are a more suitable training method due to exercise specificity.

## Introduction

Handball is a contact sport characterised by a high level of motor abilities, such as speed, explosive strength, power, and endurance (Manchado et al., 2013b). A study by Michalsik et al. (2014) showed that, during a female elite team handball match, high aerobic demands (~80% of maximum oxygen uptake [VO_2max_]) interspersed with very brief time periods of substantial anaerobic energy production (~1% of total effective playing time per match) were observed among players. Team handball players must coordinate their movements in running, jumping, and pushing, change of direction, and team handball-specific movements, such as passing, catching, throwing, checking and blocking (Michalsik et al., 2013, 2015a, 2015b, 2015c).

According to Michalsik (2021a), it is not possible for elite team handball players to always function at the top level throughout the competitive season. Handball training must be periodized for players to perform as well as possible in the most important competitions and matches during the competitive season. Physical training should be organised with preplanned, systematic variations in the specificity, intensity, and volume of training at different times or cycles within the overall training programme for the season (Issurin, 2010; Michalsik, 2021a). Training programmes for elite team handball players should use periodisation to divide training into an off-season, a preseason and a postseason, for example, with each period focussing on different goals. During the preseason, training emphasises physical fitness improvements, whereas during the in-season, the emphasis is mainly on tactical and technical preparation while maintaining physical fitness (Cherif et al., 2012).

In recent years, the small-sided games (SSG) training method has become the focus of scientific research due to its ability to develop physical abilities together with sport-specific tactical and technical skills (Dello Iacono et al., 2016). SSG training has become a great success, especially in soccer (Praça et al., 2022) and other team sports, in which it offers many practical advantages (such as facilitating the development of technical and tactical skills, as well as improving the endurance of team sport players) that have led to its popularity as a training modality (Halouani et al., 2014). The primary benefits of SSGs are that the games can replicate the movement patterns, physiological demands, and technical requirements of a competition (Dello Iacono et al., 2015), while also requiring players to make decisions under conditions of pressure, stress, and fatigue. In addition, SSG training is expected to increase players’ compliance and motivation compared to traditional training techniques, since it is perceived as a sports exercise that maximises ball training time (Buchheit et al., 2009).

A handball SSG-based training programme has been shown to be effective in improving repeated sprint ability, agility, jump height, and sprint performance (Dello Iacono et al., 2016). SSGs have also been widely used to improve match-specific aerobic and anaerobic fitness while involving the technical skills required in handball (Buchheit et al., 2009).

In our study, we focused on elite female handball players to expand the knowledge and increase the effectiveness of the specificity of the training process in handball, because it is necessary to analyse the training process under real training conditions in the preseason period. The main aim of the study was to compare the training methods of small-sided games of handball and continuous endurance training and their effects on handball players' fitness.

## Methods

### Participants

Eighteen elite female handball players who were competing in the first international league (Women’s Handball International League [WHIL]) for female players in the Czech Republic voluntarily participated in the study.

Players were divided into three groups according to their playing position (pivots [n = 3], wings [n = 5], and backs [n = 10]). They were then randomly assigned from each playing position group either to the SSG group (SSGg; pivots n = 1, wings n = 3, backs n = 5) or to the running group (RG; pivots n = 2, wings n = 2, backs n = 5). Both groups continued regular preseason training for six weeks. The SSGg (age: 21.22 ± 3.03 years, body height: 174.22 ± 5.97 cm, body mass: 68.21 ± 9.37 kg, HR_max_: 202.3 beats/min^-1^) also performed SSG training, and the RG (age: 23.78 ± 3.77 years, body height: 170.78 ± 7.43 cm, body mass: 70.6 ± 13.98 kg, HR_max_: 199.2 beats/min^-1^) performed endurance running (Continuous Endurance Running Training; CERT). None of the players participating in the study suffered from COVID-19. Written informed consent to participate in the study was obtained from each player in accordance with the ethical standards of the Helsinki Declaration. The study was approved by the Ethics Committee (no. 52/2014) of the Faculty Physical Culture at the Palacky University in Olomouc.

### Design and Methods

#### Training Variables of Both Groups before and during the Intervention

Three weeks before the pretests, players followed the same physical training programme (without the ball), which they had to perform individually and not under the supervision of their club. Training could not be supervised and controlled; therefore, no training data were collected. It was a four-day per week programme that included coordination, speed, strength and endurance training.

During the six weeks in the preseason, players attended two training sessions per day, resulting in 10 training units per week (60 training sessions in total), and they played nine scrimmage matches during which players were regularly substituted. During the monitoring period, players had four weekends free and two weekends playing in tournaments. The general training variables were the same for both groups studied, and for the specific training variables, players of both groups had the same values; only for aerobic and anaerobic endurance did they differ due to the experimental intervention (Table 1).

**Table 1:** Training variables during the intervention in both groups.

General training indicators of both groups	
Number of load days (including tournament days)	34
Number of training sessions (number)	60
Total load time (hours) (including matches)	99
Number of matches in tournament (number)	6
Number of free days	8
**Specific training indicators of both groups**	Number of hours
Warm-up	20
Aerobic endurance	15 RG 9 SSGg
Anaerobic endurance	3 RG 9 SSGg
Strength	12
Speed	2
Coordination	2
Individual attacks actions	2
Individual defensive actions	2
Attacking combinations	6
Defensive combinations	4
Attacking systems	7
Defensive systems	6
Training game	6
Preparatory match (2 x 30 min)	3
Total load hours for each group	90

#### Handball Small-Sided Games Training (SSGT)

SSGs were played by 4 versus 4 + 1 floater players. A floater player alternatively participated in offense or defense. This player was able to create situations of numerical superiority in the attack. One SSG was played for four minutes, and four SSGs were played in a row. The rest interval between each SSG was three minutes. Thus, in one training session, there were four SSGs of four minutes each, with a 3-min rest interval between games. A similar load and rest model was used in other team sports, such as those used in the studies by Brandes et al. (2012) and Bělka et al. (2021). The heart rate was recorded using Polar Team Pro System monitors (Polar Electro Oy, Kempele, Finland).

#### Continuous Endurance Running Training (CERT)

Players ran at an individual pace, always in the same outdoor section on a flat asphalt surface without any terrain elevation for a period of 30 min, and they had to maintain an average heart rate intensity higher than 80% of HR_max_, which was monitored by the coach using a tablet with Team Polar software installed. The HR and distance travelled were measured by sports systems equipped with the GPS (Polar Electro Oy, Kempele, Finland).

Both interventions supplemented a normal handball training programme during the preseason period. The six-week preseason period of training was assumed to be standard handball training appropriate for this sports level, as confirmed by Ganados et al. (2008). Intervention training sessions always lasted 90 min. The first 20 min consisted of a warm-up characterised by passing between players, followed by a 30-min tactical training session. This session was followed by SSG and CERT interventions. Each week, players had SSG and CERT interventions twice during the microcycle. The SSGg played 12 x SSGs, and the RG performed 12 training sessions of CERT during the monitored 6-week period, similarly as in the study of Eniseler et al. (2017). These training sessions always took place on Tuesdays and Thursdays during the afternoon in the last 40 min of the training unit. The SSGg played SSGs, while the RG performed a continuous run. After 30 min, the groups joined together for stretching exercises. Players completed the same training sessions over 6 weeks. The division into groups was based on the players’ game positions; thus, every game position was represented in each group. Goalkeepers were excluded from the research due to their specific positions and were only needed in the SSGg for SSGs.

#### Fitness Testing

Fitness tests (pretest/posttest) were performed prior to the six weeks of intervention and one week afterwards. The pretest was on the 13^th^ of July, 2020, and the posttest was on the 31^st^ of August, 2020, always performed between 5 and 6.30 pm. Both fitness tests were performed in the afternoon based on the findings of Mheni et al. (2017), who reported that short-term maximal physical performance related to team handball was better in the afternoon than in the morning for female handball players. All participants performed a 20-min warm up before the fitness tests. The order of fitness tests in sequence was the 20-m and 30-m sprints, the Modified Agility T test, the Standing Long-Jump Test, a throw from 7 m, and the Yo-Yo Intermittent Recovery Test level 1. The tests were chosen to determine the overall fitness level of female players: speed (20-m and 30-m sprints), agility (modified agility T test), explosive upper limb strength (throw from 7 m), explosive lower limb strength (Standing Long-Jump Test) and intermittent endurance running capacity (YoYo IR1), as recommended for women. All tests were part of other studies targeting the fitness of handball female players. All players executed these fitness tests in the past. Before each test, players were informed about the process and the objective of the test. Before the main trials, players completed an additional test trial, except for the YoYo IR 1 test, which they could complete once, but they were given a practice run of the first two rounds. Testing always occurred in the afternoon in a sports hall on the handball field with a synthetic floor (lobadur).

**20-m and 30-m Sprint Tests**. In the 30-m sprint test, each player performed a sprint as fast as possible as in the study by Chaouachi et al. (2009). To measure sprinting time, we employed three pairs of light beams (Brower Timing System TCi-System, Draper, USA) placed at the 0-, 20-, and 30-m marks of the testing distance. Each player had to repeat the sprint test twice (with a 2-min rest interval between tests). The fastest 20- and 30-m sprint times were used for analysis.

**Modified Agility T Test (MAT)**. The MAT was used as a relevant test for team handball players to determine their speed of changing direction, such as forwards sprinting, left and right shuffling, and running backwards (i.e., the uppercase ‘T’ shape represents the displacement diagram of the participant during the test; Mhenni et al., 2017). The MAT is described in detail in a study by Simão et al. (2009). The score recorded for this test was the fastest time from the two trials, with 3-min rest intervals between trials. MAT performances were recorded using electronic timing gates (Brower Timing System TCi-System, Draper, USA).

**Standing Long-Jump Test (SLJT)**. Each player performed a series of horizontal jumps, as described in a study by Chaouachi et al. (2009). Participants were allowed three trials for each jump, with the longest distance jumped recorded for further analysis.

**Ball-Throwing Velocity**. The test was chosen because we wanted to determine whether SSG training has an effect on the explosiveness of the upper limbs, and is described in a study by Saavedra et al. (2018). Each player executed three standing throws with a 2-min rest interval between trials. The greatest velocity throws were used for subsequent analysis. The ball speed for the three throws was measured with a calibrated radar (Multi Sports Personal Speed Radar, Net Playz, Tchaj-wan), which was placed in the middle of the goal.

**Yo-Yo Intermittent Recovery Test Level 1**. According to Michalsik et al. (2021b), the Yo-Yo IR1 test examines intermittent endurance running capacity and is relevant due to its team sport-specific character. Converted to team handball, it is all about measuring the ability of players to maintain a high playing pace throughout the entire game, in which players, e.g., repeatedly must perform fast breaks and fast retreats (Michalsik et al., 2021b). The test was performed as in the study by Michalsik et al. (2021b).

#### Statistical Analysis

Statistical analysis was performed using the Statistica data analysis software system, version 13 (Stat Soft, Inc., Tulsa, OK, USA). Descriptive statistics were calculated for all variables and are reported as the mean ± SD. The Kolmogorov-Smirnov test and the Levene’s test were used to verify the normality and homogeneity of variances. To compare preintervention values, the nonparametric Mann-Whitney test was used with Cohen’s *d* effect size (ES). Friedman’s ANOVA of repeated measures was used to determine the significance of pre- and postintervention differences of observed variables with a η^2^ effect size. All data analyses were performed using IBM SPSS software (version 25, IBM, Armonk, NY, USA) with alpha ≤ 0.05.

## Results

The mean heart rate (HR) of players during SSGs was 176.8 ± 6.6 beats/min resp. 87.4 ± 3.3% HR_max_. The mean HR of players during CERT was 160.7 ± 12.7 beats/min resp. 80.7 ± 0.06% HR_max_. Players covered a distance of 4980.3 ± 180.1 m.

Pre- and posttest means ± SDs and differences among female handball players were measured in the six-week preseason SSG and CERT training for sprints, jumps, shots, and intermittent endurance running. All data were normally distributed, and no violation of homogeneity of variance was found. The SSGg and RG showed no significant differences at baseline for any variable (Table 2).

**Table 2:** Comparison of pretest values between the RG and the SSGg.

	Z	U	*p*
20 m (s)	−1.29	25.50	0.20
30 m (s)	−1.55	22.50	0.12
t-test (s)	−1.68	21.00	0.09
Standing Long-Jump Test (m)	−1.72	14.00	0.06
Shot 7 m (km•h^-1^)	0.44	35.00	0.65
Yo-Yo IR1 (m)	1.24	26.00	0.21

Note: Z – score, U – U statistics, p – significantly different values

The results show that both groups improved in particular fitness tests during the study period (Table 3). For the SSGg, there was a significant improvement in the T test (*p =* 0.034), ball-throwing velocity (*p =* 0.019), and the Yo-Yo IR1 test (*p =* 0.003). The RG showed a significant improvement in jumping (*p =* 0.049), ball-throwing velocity (*p =* 0.003), and the Yo-Yo IR1 test (*p =* 0.003).

**Table 3:** Comparison of pretest and posttest by the SSGg and the RG.

	SSGg			RG		
Performance Test	pre	post	ES	pre	post	ES
20 m (s)	3.24 ± 0.16	3.19 ± 0.13	0.3	3.35 ± 0.17	3.30 ± 0.17	0.2
30 m (s)	4.60 ± 0.20	4.54 ± 0.16	0.1	4.76 ± 0.23	4.68 ± 0.27	0.3
*t*-test (s)	5.95 ± 0.22	5.81 ± 0.19*	0.5	6.2 ± 0.32	6.17 ± 0.28	0.1
Standing Long-Jump Test (m)	2.19 ± 0.16	2.26 ± 0.13	0.3	2.00 ± 0.12	2.04 ± 0.14*	0.3
Shot 7 m (km•h^-1^)	70.11 ± 8.96	78.56 ± 6.67*	0.6	68.22 ± 5.87	73.11 ± 4.01*	0.9
Yo-Yo IR1 (m)	840.0 ± 207.85	1182.22 ± 285.03*	0.9	737.78 ± 226.37	1071.11 ± 423.69*	0.9

Note: Significant difference from pretraining values, * p < 0.05, ES – effect size; SSGg – SSG group; RG – running group; pre – pretest; post – posttest

Both groups had significant pre- and posttest differences in ball-throwing velocity and the YYIR1 (*p <* 0.05).

The SSGg only featured significant differences between pre- and posttraining in the T test (*p <* 0.05). The T test had medium ES differences between the pre- and posttests (ES = 0.5). The RG showed small ES differences between the pre- and posttests in the T test (ES = 0.1). The RG only showed significant differences between pre- and posttraining values in the jump test (*p <* 0.05). The jump test showed small ES differences for the RG between the pre- and posttests (ES = 0.3), as did the SSGg.

In the 20-m sprint, there were small ES differences between the pre- and posttests (ES = 0.3 and 0.2, respectively) in both groups. The same was true for the 30-m results, with small ES differences between the pre- and posttests (ES = 0.1 and 0.3, respectively) for both the SSGg and the RG.

## Discussion

This study is the first to document differences in the effects of handball SSG training versus the continuous running method on the fitness level of female players during the preseason in handball. For the female team that we studied, a more traditional periodisation of training (from a high volume and low intensity phase during the preparatory periods to a low volume and high intensity phase when approaching the competitive periods) was applied, as reported by Simão et al. (2012).

Our results indicate significant differences in the fitness tests of female players using these two training methods. However, we must be cautious with the conclusions because there are other factors (e.g., menstrual cycle, hydration, quality and quantity of sleep, mental mood of players on testing days, etc.) that might have influenced our results that we did not observe. Nevertheless, our results suggest possible advantages of using SSGs in handball training. Our results demonstrate that small-sided games and handball training in the pre-competitive period, can be considered a useful tool for the improvement of agility, throwing and endurance running. Our findings are in line with other research (Dello Iacono et al., 2015, 2016; Buchheit, 2009).

In linear sprints up to 30 m, there was no statistically significant improvement in any of the monitored groups. Jurišić et al. (2021) obtained the same statistically non-significant results in the 30-m sprint in their study for the SSG intervention group. Developing speed is one of the most difficult tasks of training, because speed is largely influenced by genetics, more precisely by the ACTN3 RR genotype, which is associated with speed performance (Varillas-Delgado et al., 2022). Improvements in sprinting ability are largely due to neuronal adaptations (Behm and Sale, 1993), including a selective and greater activation of motors units, and better synchronization (Hammami et al., 2019). Speed of limb movement, explosive strength, running speed and agility show peak development at peak height velocity, on which neuromuscular adaptation is also dependent (Philippaerts et al., 2006). For these reasons, we believe that none of our intervention training methods (SSGT, CERT) could have an effect on speed development because they did not meet this principle.

The results of the pretest and posttest of the 20-m speed test from our study are comparable to the results of a study by Pereira et al. (2018), in which players ran 20 m in 3.25–3.29 s. In addition, results similar to those of our study were found by Saavedra et al. (2018) and Wagner et al. (2019) in studies in which players covered a distance of 30 m in 4.57 ± 0.16 s resp. 4.7 ± 0.12 s. Both of those studies used elite female players as their samples. For this reason, we believe that female players did not have low initial speed performance. The SSG group improved by 1.5% between the pre- and posttest, similar to the study by Jurišić et al. (2021), in which there was an improvement of 1.68% for the SSG group in the preseason period.

Acceleration and fast changes of direction are parts of handball training and matches. The initial speed and acceleration of players are important information for coaches, as well as their changes in speed, direction, and stopping. As in another intervention study on handball players (Hammami et al., 2019), there was a significant improvement in the modified T test in the SSGg in our study. One of the aspects that could have influenced this result was that players who played SSGs were much more likely to make specific movements, such as those that are part of a modified T test (changes of direction, running backwards, etc.). In contrast, the RG did not improve in the T-test, and one possible explanation for these different results could be that CERT did not include any form of exercise where athletes had to change direction, as Hammami et al. (2019) also state similarly. In the MAT, the movements with direction changes are predetermined, and it is assumed that the learning effect can also influence the results. In our study, players had performed the MAT repeatedly in the past, thus we believe that the effect of the learning rate was minor on the results, and a significant difference would likely have occurred in both groups, while this was not the case. Performance (5.95 ± 0.22–6.17 ± 0.28 s) similar to those of players in our study in the modified T test was found in female handball players in a study by Mhenni et al. (2017) (6.05 ± 0.27 s and 5.65 ± 0.07 s, respectively), in which the morning and afternoon performance of players in various fitness tests was monitored. Therefore, we believe that our baseline results from the MAT in the pretest are comparable to those of other studies.

In the Standing Long Jump test (SLJT), which focused on dynamic lower limb strength, there was a statistically significant improvement in the RG. In our opinion, this improvement is not attributable to the intervention, but rather to the lower level of the initial tests, and possibly, to the additional training process aimed at developing dynamic lower limb strength, which was common in both groups during the follow-up period. Our results (2.04 ± 0.14–2.26 ± 0.13 m) in the SLJT are higher than those of another study (Labib, 2013) in which handball players achieved a performance of 2.02 ± 0.04–2.1 ± 0.05 m.

In our study, preseason handball training led to a significant increase in throwing velocity. Both groups showed a significant improvement in throwing speed between the pretest and posttest. We believe that the increase in strength was again due to lower input values, normal handball training, and supplemental strength training aimed at developing explosive strength, which was common to both groups in the intervention period. In contrast, we believe that the nature of the intervention (SSGT), which included elements related to ball handling (passing and shooting) to increase explosive upper limb strength, had a partial effect on the increase in upper limb strength in the SSG group players, similar to the handball match according to Granados et al. (2007). In their study, Gorostiága et al. (2005) described significant relationships between changes in the body fat percentage and changes in lower and upper limb muscle performance. This suggests, according to Granados et al. (2008), that female handball players playing more minutes during matches are likely to achieve significant increases in fat-free mass (FFM) and the ability to move quickly the upper limbs, compared to those who play fewer minutes during matches. Increases in FFM can be considered a positive adaptation in elite female handball players, because elite female handball players have 10% more FFM than lower level players (Granados et al., 2007), and because higher FFM produces greater power and speed when throwing (Van Den Tillaar and Ettema, 2004).

In the YYIR1 test, both study groups improved significantly. Over the entire follow-up period, the RG covered an average of almost 60 km, and the intervention was 1/3 of the time for the development of general endurance during this follow-up period (Table 2). Despite the relatively low running intensity and duration, we believe that the RG group was at least partially affected by this method in improving its performance in the YoYo IR1 test as in a study by Soylu et al. (2021). According to Manchado et al. (2013a), more attention should be given to high-intensity endurance training when planning an entire training season, since aerobic capacity is important to maintain high performance for 60 min of play. In many sports, for example, team ball games, such as soccer and team handball, exercise is intermittent, and physical performance is related to the players’ ability to repeatedly perform intense exercise throughout the entire game (Michalsik et al., 2021b). High Intensity Interval Training, which includes SSGs, is one method that is commonly prescribed within the Team Based Field Sport Athletes (TBFS) setting, with increased VO2peak and glycogen utilisation among a host of physiological benefits that are derived with the correct implementation (Milanović et al., 2015). This is in line with several other studies that illustrate an increased aerobic contribution with an increasing quantity of sprints, confirming the hypothesis above that anaerobic glycolysis and aerobic metabolism are the most important energy sources for TBFS athletes (Buchheit and Laursen, 2013). Establishing the gold standard for enhancing these energy systems is thereby critical to ensure the improvement and subsequent performance of TBFS athletes for their sports (Bishop, 2021). Our results for both study groups from the YYIR1 were lower (1,182.22 and 1,071.11 m, respectively) than the results from a study by Michalsik et al. (2014), although our study also included elite handball players. In another study by Michalsik et al. (2021), elite female handball players covered a distance in the YYIR1 between 1,330 and 1,660 m; thus, we must conclude that the results in the YoYo IR1 test of elite players from Denmark are at a completely different level. An explanation of these results would require a detailed analysis of the training process of players in Denmark and the Czech Republic.

In our opinion, this study was influenced by several factors, such as the performance level of players, the quality and quantity of players' sport and fitness training, and the pandemic measures of COVID-19 in the Czech Republic; or as Michalsik et al. (2014, 2021) reported in their studies, the results of fitness tests performed in the first days after the summer break (at the beginning of the preseason) tend to be lower than the results of tests performed during the competition part of the season, when players are already used to movements similar to team handball, such as acceleration, deceleration and change of direction.

Based on our results, we may conclude that the SSG training method is a more appropriate means of developing handball players' fitness in the preseason compared to the CERT method, because the SSGT method had a positive effect on the MAT, ball throwing speed, and YoYo IR1 scores, and the CERT method had a direct effect only on YoYo IR1 scores. Another benefit of the SSGT method is its specificity in terms of the simultaneous development of tactical and technical skills in handball. We believe that the inclusion of this method in the training process at least twice per week in the preseason is a suitable means for increasing general fitness of female handball players.

## Limitations of the Study

The lack of supervision and control of preintervention physical training, and the absence of a familiarisation test round for all the tests (pretest round) might have had a significant influence on preliminary test results and the size of potential improvements during the study period when comparing pretraining to posttraining test results. Without a normal/suitable amount of physical training and one or more pretest rounds prior to the intervention period, improvements in test results could have been strongly influenced by the normal on-court team handball training and the learning effect in the applied tests, rather than exclusively the effects of the specific training intervention in the two groups. We also perceive the lack of a control group as a limitation of our study. However, the absence of a control group is common in studies comparing training interventions. Another limitation could have been the order of the tests in the actual testing, which could have negatively affected our results for tests that were performed at a later stage, although the logical order of the tests was followed, which was similar in other studies. Another limitation of the study might have been the use of the YoYo IR1 field test designed for female handball players. Its intermittent nature might have affected performance of female players in the RG, but there was still an improvement in this group. The lower values for some of the pretest results could have affected our results. The results of the 7-m throw test should be interpreted with caution since the validity of this test is unknown, although it has been reported in several handball studies.

## Conclusions

In conclusion, based on the results, the SSGT method had a positive effect on the MAT, ball-throwing velocity and YoYo IR1 results, and the CERT method had a direct effect only on the YoYo IR1 results.

According to our findings, we believe that the CERT method used in this study is not a suitable means of developing preseason fitness in female handball players. In contrast, we found that, in modern handball, the SSGT method is more appropriate for the development of fitness, except for the development of speed, in the preseason period.
